# Deep learning algorithm enables automated Cobb angle measurements with high accuracy

**DOI:** 10.1007/s00256-024-04853-7

**Published:** 2024-12-17

**Authors:** Daichi Hayashi, Nor-eddine Regnard, Jeanne Ventre, Vincent Marty, Lauryane Clovis, Ludovic Lim, Nicolas Nitche, Zekun Zhang, Antoine Tournier, Alexis Ducarouge, Andrew J. Kompel, Chadi Tannoury, Ali Guermazi

**Affiliations:** 1 Department of Radiology, Chobanian and Avedisian School of Medicine, Boston University, Boston, MA USA; 2 Department of Radiology, Tufts Medical Center, Tufts University School of Medicine, 800 Washington Street, #299, Boston, MA 02111 USA; 3 Réseau Imagerie Sud Francilien, Lieusaint, France; 4 Gleamer, Paris, France; 5 Department of Orthopedic Surgery, Chobanian and Avedisian School of Medicine, Boston University, Boston, MA USA; 6 Department of Radiology, Veterans Affairs Boston Healthcare System, West Roxbury, Boston, MA USA

**Keywords:** Scoliosis, Deep learning, Cobb angle, Radiograph

## Abstract

**Objective:**

To determine the accuracy of automatic Cobb angle measurements by deep learning (DL) on full spine radiographs.

**Materials and methods:**

Full spine radiographs of patients aged > 2 years were screened using the radiology reports to identify radiographs for performing Cobb angle measurements. Two senior musculoskeletal radiologists and one senior orthopedic surgeon independently annotated Cobb angles exceeding 7° indicating the angle location as either proximal thoracic (apices between T3 and T5), main thoracic (apices between T6 and T11), or thoraco-lumbar (apices between T12 and L4). If at least two readers agreed on the number of angles, location of the angles, and difference between comparable angles was < 8°, then the ground truth was defined as the mean of their measurements. Otherwise, the radiographs were reviewed by the three annotators in consensus. The DL software (BoneMetrics, Gleamer) was evaluated against the manual annotation in terms of mean absolute error (MAE).

**Results:**

A total of 345 patients were included in the study (age 33 ± 24 years, 221 women): 179 pediatric patients (< 22 years old) and 166 adult patients (22 to 85 years old). Fifty-three cases were reviewed in consensus. The MAE of the DL algorithm for the main curvature was 2.6° (95% CI [2.0; 3.3]). For the subgroup of pediatric patients, the MAE was 1.9° (95% CI [1.6; 2.2]) versus 3.3° (95% CI [2.2; 4.8]) for adults.

**Conclusion:**

The DL algorithm predicted the Cobb angle of scoliotic patients with high accuracy.

## Introduction

Cobb angle measurements on spine radiographs are routinely performed by radiologists, orthopedic surgeons, and pediatricians treating patients with scoliosis. Although it is a relatively simple task, performing the Cobb angle measurement repeatedly on a large number of radiographs can become mundane and burdensome for human readers. Deep learning (DL) can help in such a circumstance by semi-automating the angle measurements, providing it can perform the task accurately and reliably.

DL-assisted automated or semiautomated Cobb angle measurements have been described in recent literature. Attempts have been made to use DL and measure Cobb angle in both adult and pediatric populations on frontal spine radiographs [1–6] and chest radiographs [7]. Investigators in these studies used different measurements to evaluate the reliability and accuracy of the DL algorithm compared to human readers, making a direct comparison of results difficult. In general, these studies reported DL algorithms having excellent reliability in terms of intraclass correlation coefficient against human readers (ranging from 0.78 to 0.98 [4–6]) and the Pearson correlation coefficient of 0.990 [3]. Reported measurement errors of the Cobb angles were all smaller than 10° [2–7]. To help clinicians, it is desirable to develop DL-based software that can assist readers by improving operational efficiencies and diagnostic accuracy. Moreover, ideally, such software should be applicable to both pediatric and adult patients. The aim of our study is to evaluate the performance of a DL algorithm BoneMetrics for measurements of Cobb angles on spine radiographs.

## Materials and methods

### Data characteristics

Two datasets were created for the purpose of retrospective validation during which the performances of BoneMetrics were assessed and compared to the ground truth defined for every image included in the dataset.

**Fig. 1:**
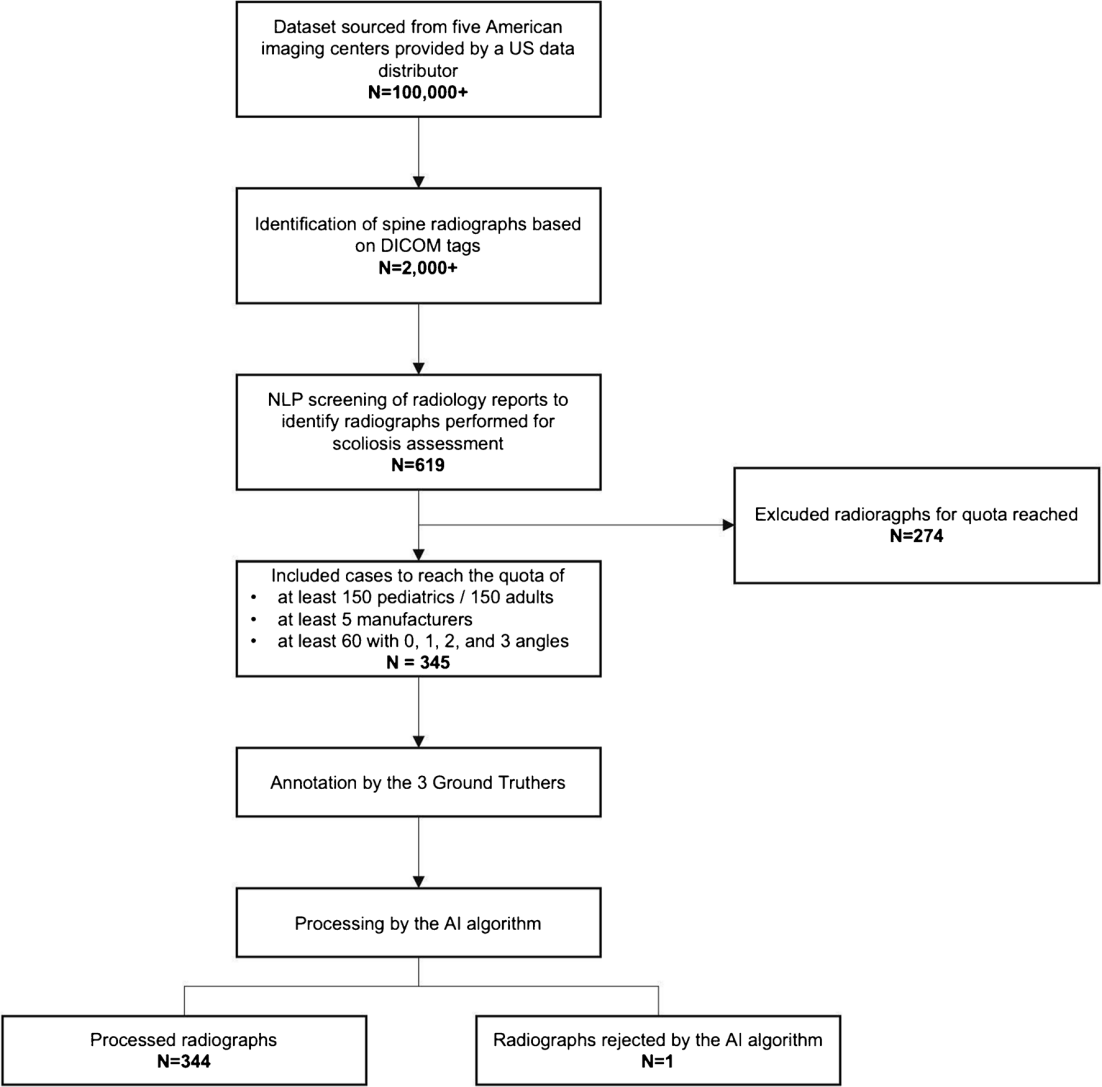
Flow chart explaining the radiograph selection process, manual annotation of the ground truth by 3 readers, and processing of the radiographs by AI algorithm for automated Cobb angle measurement

The first dataset contained data from adult patients, composed of at least 150 images from the United States. The second dataset contained data from children and adolescents, composed of at least 150 images also from the United States. The images for these two datasets were randomly sampled from an anonymized clinical dataset owned by data providers in the United States, spanning the period from September 2008 to February 2023, and originating from five different sites. The “adult (22 years or older) dataset” contained anonymized conventional frontal spine radiographs with or without scoliosis (measurable Cobb angle). Similar radiographs of patients aged between 2 and 21 years were included in the “child/adolescent dataset.” The exclusion criteria for the radiographic images in these datasets include those with poor image quality, those with embedded measurements, and those with a wrong radiographic view. We included cases to reach the quota of at least 150 pediatric patients and 150 adult patients, at least 5 manufacturers, and at least 60 cases with 0, 1, 2, and 3 measured angles (Fig. 1).

### Ground truth definition

The ground truth for Cobb angle measurements was defined by three expert readers (two senior musculoskeletal radiologists and one senior spine orthopedic surgeon (Fig. 1). As the first step, for each image, the readers determined the following independently: the number of Cobb angles larger than 7° annotated on the image (from zero up to 3 Cobb angles per patient); the value of each Cobb angle annotated (if any); the type of vertebra (cervical, thoracic, or lumbar) with the largest curvature used for the measurement of each Cobb angle annotated (if any). Cobb angle measurements were performed using proprietary software (Kili). The following classification of scoliosis was used: proximal thoracic (apices at T3, T4, or T5); main thoracic (apices between T6 and T11); thoraco-lumbar/lumbar (apices between T12 and L4).

After the first step, an automatic global review of all measurements submitted by the ground truth readers was performed. The ground truth for each image was defined when the number of Cobb angles is the same for at least two readers; AND (if any) the type of vertebra (cervical, thoracic, or lumbar) with the largest curvature used for the measurement of each Cobb angle is the same for at least two readers who agreed on the number of angles; AND (if any) the value of the Cobb angles identified by the readers is within 8° of difference [8, 9] for at least two readers who agreed on the number of Cobb angles, and on their type of vertebra (cervical, thoracic, or lumbar) with the largest curvature. Then, the ground truth was defined as the mean values of the angles measured by the two most concordant annotations by the readers. If all 3 annotations were concordant, then the ground truth was defined as the mean values of the three angle measurements.

**Fig. 2:**
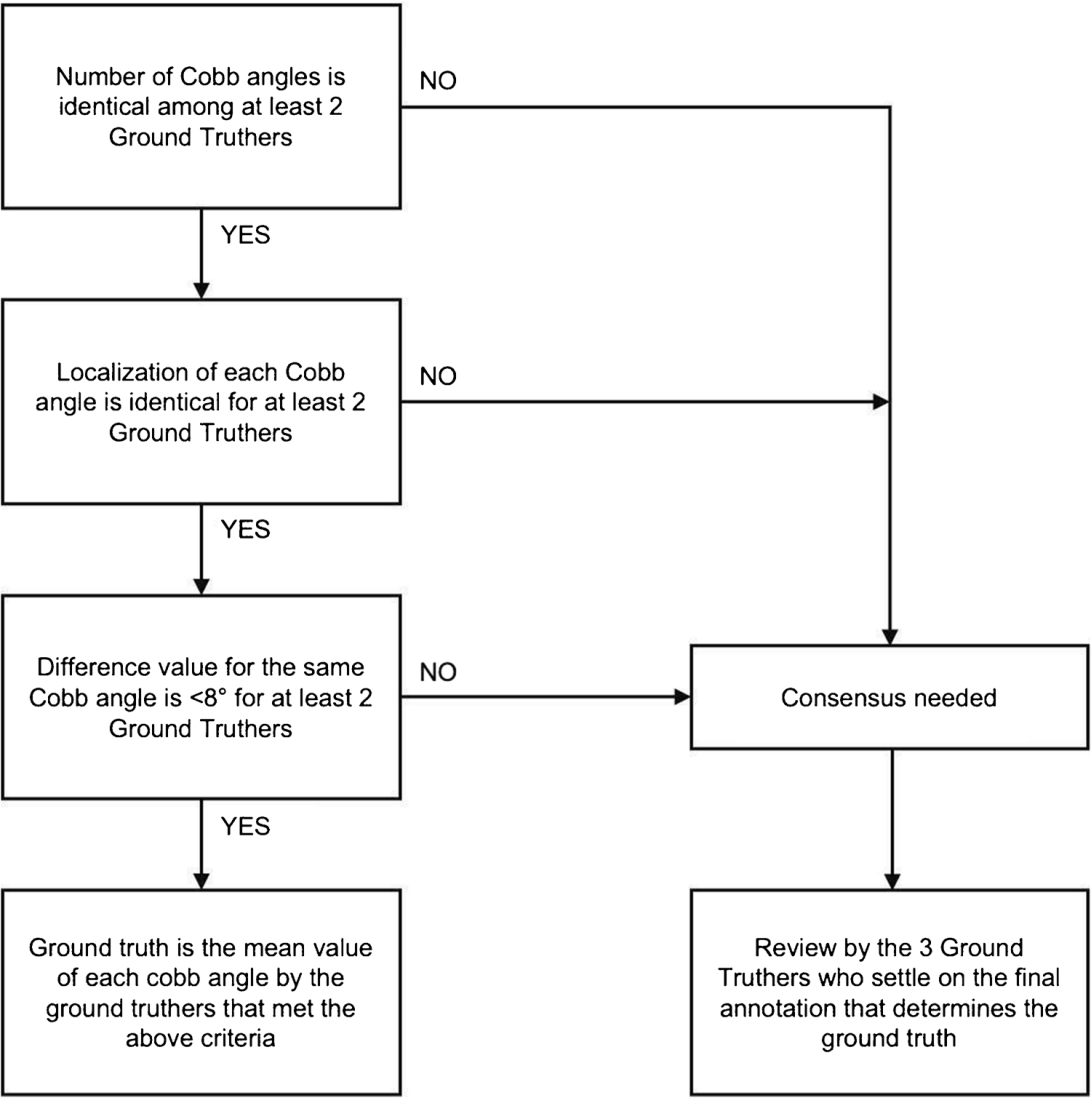
Flow chart for consensus reading by three expert readers

In certain cases, the ground truth could not be defined from the initial annotations by 3 readers, and the image had to go through the reconciliation process (Step 3). Namely, if the number of Cobb angles is different among the ground truthers; OR (if any) the type of vertebra (cervical, thoracic, or lumbar) with the largest curvature used for the measurement of a Cobb angle is different between the readers; OR (if any) the value of the Cobb angles identified by the readers is more than 8° of difference for at least the two readers who agreed on the number of Cobb angles, and on their type of vertebra with the largest curvature. In the reconciliation process (Step 3), the selected images were repeatedly reviewed by the three readers to reach a consensus with regard to the number of Cobb angles, (if any) the value of each Cobb angle annotated, and (if any) the type of vertebra (cervical, thoracic, or lumbar) used for the measurements of the Cobb angles (Fig. 2).

### Deep learning algorithm (BoneMetrics)

BoneMetrics (v2.3.1, Gleamer, Paris, France) is a CE-marked fully automated radiological image processing software for conventional and EOS radiographs. The software includes a DL algorithm of several convolutional neural networks based on pose estimation and key point detection models. The algorithm utilizes several architectures, including a top-down model derived from Mask-RCNN architecture implemented using Detectron2, a lightweight HRNet (liteHRNet), and a bottom-up approach. The algorithm also incorporates image augmentation techniques, such as horizontal flips, cropping, scaling, and rotation, to improve robustness and generalizability during training. The algorithm evaluates each predicted key point with a confidence score from 0 to 100, and only key points with scores exceeding the threshold of 50 are employed in calculating a set of measurements. The AI is automatically triggered by any acquisition of a full-spine radiograph in the picture archiving and communication system (PACS) and requires only one critical information in the DICOM fields, which is the patient’s age.

The algorithm calculates Cobb angles between C7 and L5, and the three maximum curvatures are then returned in a secondary capture. The algorithm filters out curvatures below 7° to exclude clinically insignificant measurements. To ensure the model’s accuracy and generalization, it was trained on a dataset of 5000 + annotated radiographs from more than 20 public and private settings from multiple patient populations and geographies. Data in the development dataset was selected from diverse sources with a balance of different criteria: radiography system manufacturers, pathology (e.g., severe scoliosis, fracture of a vertebral endplate), X-ray acquisition type, quality (sub-optimal images included), patient age, and body mass index.

The development dataset was annotated by a pool of 10 trained radiologists and radiographers, and the quality of annotation was monitored by a senior musculoskeletal radiologist with 14 years of experience. There is no overlap of patient or image between the study dataset and the AI development dataset.

After determining the ground truth, each image of the two datasets was then processed by the BoneMetrics algorithm. For each image, the results of the algorithm of BoneMetrics were compared with the ground truth.

### Statistical analysis

To describe the dataset, summary statistics were computed for patient characteristics such as sex, age, and deformities. To assess the performance of BoneMetrics, the mean absolute error (MAE) was used as an endpoint. The MAE is a measure of errors between paired observations expressing the same phenomenon, i.e., in this study, the MAE is the mean difference between the Cobb angle values provided by the DL algorithm and by the ground truth. The MAE values were reported with a 95% bootstrap confidence interval (CI). The primary endpoint was the MAE for the values of Cobb angle of major scoliotic curves (i.e., the Cobb angle with the largest curvature in a patient). The secondary endpoint was the MAE for the values of the Cobb angle with minor curves (i.e., all Cobb angles other than the largest curvature representing the primary endpoint). Regarding the primary endpoint, an acceptance criterion was chosen using the findings of Gstoettner et al. [10] who documented a mean interobserver variability of 6.34° in their study. Thus, we decided to set the acceptance criteria to an MAE of 6.34° for both endpoints. Meeting this threshold means that BoneMetrics is a tool that can be used in clinical settings for Cobb angle measurement. Differences between subgroups were assessed using the Mann–Whitney *U* test, and statistical significance was set at *p* < 0.05. ICC was also computed to evaluate the agreement between the ground truth and the DL algorithm using the two-way random effects model with absolute agreement [11]. Four levels of reliability were defined based on the classification of ICC values: excellent, ICC ≥ 0.9; good, 0.9 > ICC ≥ 0.75; moderate, 0.75 > ICC ≥ 0.5; poor, ICC < 0.5.

## Results

**Fig. 3:**
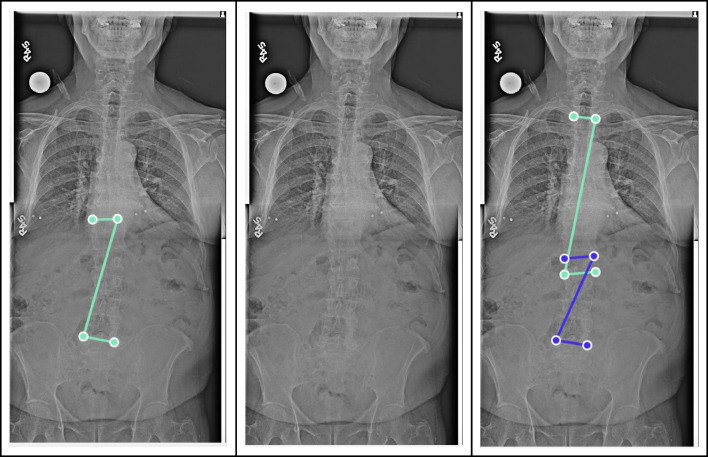
Annotated full spine radiograph for a case with a discrepancy in Cobb angle measurements among the three ground truth annotators. The image illustrates the annotated end vertebrae by each independent reader and highlights the variation in their measurements. This case required a consensus review to resolve the differences in the number of annotated Cobb angles, to determine the ground truth for the DL comparison

A total of 345 patients were included in the study (age 33 ± 24 years, 221 women), and 274 patients were excluded due to reaching the quota of at least 150 adults and 150 pediatrics, 5 distinct radiography system manufacturers, and 60 patients with 0, 1, 2, and 3 angles each. The dataset resulted in 179 pediatric patients (< 22 years old) and 166 adult patients (22 to 85 years old). For Cobb angle annotation and measurements, the distribution was as follows: 67 (19%) had no curvature with a Cobb angle > 7°, 74 (21%) had a single curvature with a Cobb angle > 7°, 118 (34%) had two curvatures, and 86 (25%) had 3 curvatures. The majority of patients had either no scoliosis (38%) or mild (10 up to 20°) scoliosis (34%), and the remaining had moderate (20 up to 40°) (20%) and severe (≥ 40°) (7%) scoliosis. Demographic characteristics of the included patients are summarized in Table 1. Following the process of ground truth determination, a total of 53 cases were reviewed in consensus (Fig. 3).

**Table 1:** Demographic characteristics of the patients

	Patients
Total number of patients	345
Number of radiography system manufacturers	5
Age, mean ± SD [range] years	33 ± 24 [4–85]
2–12 years old	81 (23%)
13–21 years old	98 (28%)
22–39 years old	35 (10%)
40–59 years old	65 (19%)
60–79 years old	59 (17%)
≥ 80 years old	7 (2%)
Sex	
Number of women (%)	221 (64%)
Number of men (%)	124 (36%)
Deformities	
No angle (%)*	67 (19%)
1 angle (%)	74 (21%)
2 angles (%)	118 (34%)
3 angles (%)	86 (25%)
No scoliosis (< 10°)^#^	132 (38%)
Mild scoliosis (10 up to 20°)	119 (34%)
Moderate scoliosis (20 up to 40°)	70 (20%)
Severe scoliosis (≥ 40°)	24 (7%)
Angles: total number of angles	568
Mean number of angles per patient	1.6
< 10°	154 (27%)
[10–20°]	248 (44%)
[20–40°]	134 (24%)
> 40°	32 (6%)
Hardware: patients with spine hardware	35 (10%)

^*^No annotation of any Cobb angle

^#^Patients with no angle annotation or an angle < 10°

The MAE of the DL algorithm for the main curvature was 2.6° (95% CI [2.0; 3.3]) with an ICC of 0.98 between the ground truth and the DL showing excellent agreement. For minor curves, the MAE was 2.8° (95% CI [2.3; 3.3]) also with a high ICC of 0.98. For the subgroup of pediatric patients, the MAE was 1.9° (95% CI [1.6; 2.2], ICC = 0.99) versus 3.3° (95% CI [2.2; 4.8], ICC = 0.96) for adults (Table 2). Statistical analysis revealed no significant differences in the MAE of the major curve between adult and pediatric patients (*p* = 0.09). However, a significant difference was observed for the MAE of the minor curve (*p* = 0.04).

**Table 2:** Summary of the MAE and ICC for major and minor curves for all patients with stratification by age

Patients	MAE for major curve [95% CI]	MAE for minor curve [95% CI]	ICC for major curve [95% CI]	ICC for minor curve [95% CI]
All	2.6° [2.0; 3.3]	2.8° [2.3; 3.3]	0.98 [0.97; 0.98]	0.98 [0.97; 0.98]
2–21 years old	1.9° [1.6; 2.2]	2.7° [2.0; 3.5]	0.99 [0.99; 1.0]	0.98 [0.97; 0.98]
22–85 years old	3.3° [2.2; 4.8]	2.9° [2.3; 3.7]	0.96 [0.95; 0.97]	0.98 [0.97; 0.99]

The MAE was 1.8° (95% CI [1.2; 2.7], ICC = 0.96) for patients with one angle, 3.0° (95% CI [1.9; 4.5], ICC = 0.96) with two angles, and 2.6° (95% CI [2.1; 3.2], ICC = 0.99) with three angles. A significant difference in the MAE was observed when comparing patients with one angle to those with three angles (*p* = 0.003). Analytic results are summarized in Table 3. The MAE was 1.6° (95% CI [0.8; 2.8], ICC = 0.92) for patients with Cobb angles between 7 and 10° (which were annotated but not considered scoliosis). For those with mild scoliosis (10 up to 20°), the MAE increased to 2.0° (95% CI [1.6; 2.4], ICC = 0.98, *p* = 0.008). Patients with moderate scoliosis (20 up to 40°) had a higher MAE of 3.6° (95% CI [2.0; 5.7], ICC = 0.96) which was not significant compared to patients with mild scoliosis (*p* = 0.19). The MAE remained the same for patients with severe scoliosis (≥ 40°), at 3.6° (95% CI [2.6; 4.8], ICC = 0.99), indicating a higher degree of measurement error as the severity of scoliosis increases. The MAE was 1.4° (95% CI [0.8; 2.2]) for consensus cases (*N* = 53) and 2.6° (95% CI [2.0; 3.3]) for non-consensus cases (*N* = 292), with no statistically significant difference between the two groups (*p* = 0.29).

**Table 3:** Summary of the MAE and ICC for major curve with stratification based on the total number of Cobb angles and on the value of the largest curvature

Patients	MAE [95% CI]	ICC [95% CI]
1 angle	1.8° [1.2; 2.7]	0.96 [0.93; 0.97]
2 angles	3.0° [1.9; 4.5]	0.96 [0.93; 0.97]
3 angles	2.6° [2.1; 3.2]	0.99 [0.99; 1.0]
No scoliosis (< 10°)	1.6° [0.8; 2.8]	0.92 [0.85; 0.96]
Mild scoliosis (10 up to 20°)	2.0° [1.6; 2.4]	0.98 [0.97; 0.99]
Moderate scoliosis (20 up to 40°)	3.6° [2.0; 5.7]	0.96 [0.93; 0.97]
Severe scoliosis (≥ 40°)	3.6° [2.6; 4.8]	0.99 [0.99; 1.0]

**Fig. 4:**
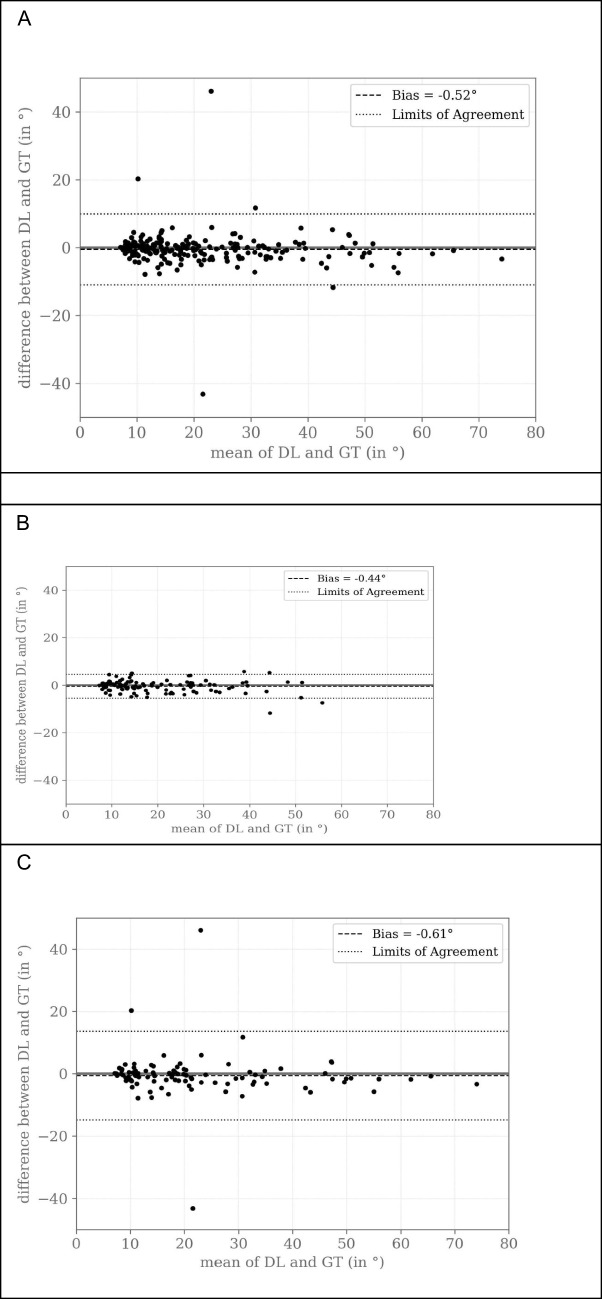
Bland–Altman plot assessing the agreement between the automatic Cobb angle measurements by the deep learning (DL) algorithm and the ground truth (GT) annotations by the three expert human readers. The difference between DL and GT measurements is plotted against the mean of DL and GT measurements. The gray solid line depicts the scenario in which DL estimates would perfectly align with the ground truth, indicating no differences between the two. The black dashed line represents the mean bias, and the black dotted lines indicate the upper and lower limits of agreement. **A** All patients, **B** pediatric patients, and **C** adult patients

Figure 4 shows the Bland–Altman plots assessing the agreement between the automatic Cobb angle measurements by the DL algorithm and the ground truth for all patients (Fig. 4A), pediatric patients (Fig. 4B), and adult patients (Fig. 4C).

**Fig. 5:**
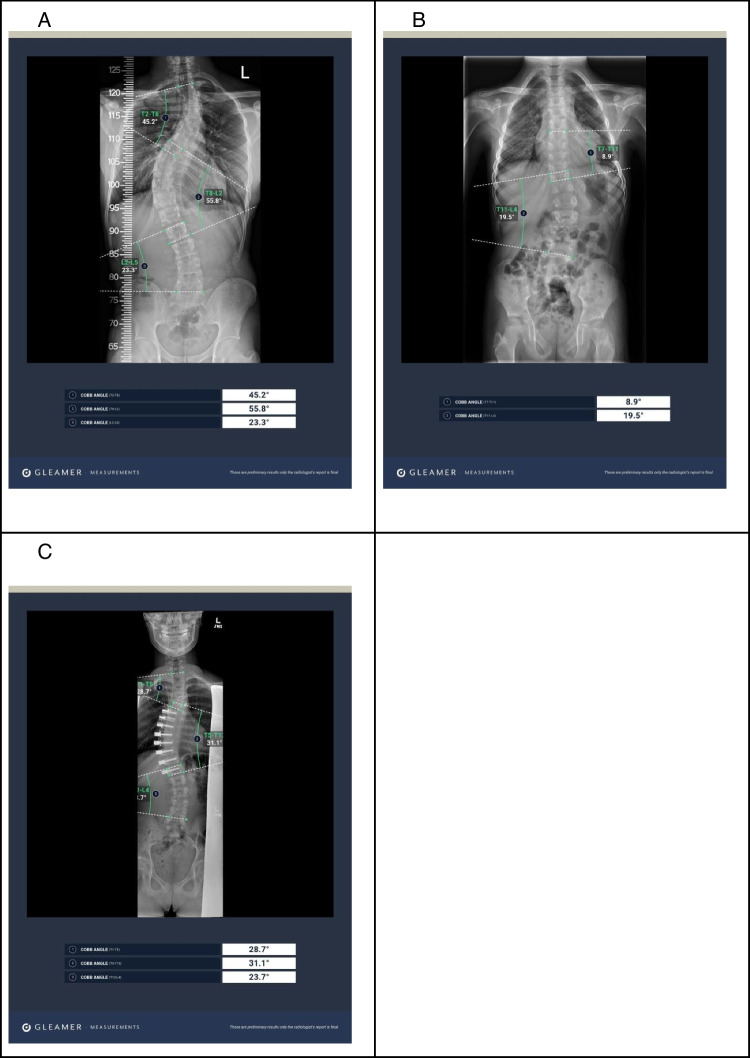
Illustrative examples of automatic Cobb angle measurements by the DL software (BoneMetrics, Gleamer) on full spine radiographs. Each image displays the spine radiograph with overlaid measurement annotations by the DL, including the detected end vertebrae used for measuring the Cobb angles and the calculated angle values: **A** 25 years old male with severe scoliosis, **B** 11 years old male with mild scoliosis, **C **14 years old girl with moderate scoliosis with thoracic vertebral screws

To provide further insights into the performance of the DL algorithm, Fig. 5 illustrates examples of automatic Cobb angle measurements on full spine radiographs across various clinical scenarios, such as pediatric patients, cases of severe scoliosis, and those involving vertebral screws.

## Discussion

In our study, automated Cobb angle measurements using a DL model (BoneMetrics) showed excellent accuracy and agreement with expert human readers for both adult and pediatric patients. Measurement errors slightly increased as the severity of scoliosis increased. The MAE values ranged from 1.8 to 3.0° in patients having between one and three scoliotic curvatures.

Agreement between expert readers and BoneMetrics was excellent with ICC of 0.98 for both main and minor curvatures. This level of agreement is similar to two previously reported studies, i.e., Horng et al. reported an ICC of 0.97 between an expert reader and their DL algorithm [1], and Sun et al. reported an ICC of 0.994 [3]. Our results showed higher accuracy compared to Berlin et al. in which ICC varied between 0.78 and 0.93 for three human readers compared to AI, depending on levels of the spine [4]. MAE of up to 3.6° (for most severe scoliosis) was noted in our study, which was lower than prior studies reporting 4.7 to 7.34° [2, 4] but higher than other studies reporting 1.7 to 2.2° [3, 5]. When compared to two other commercial AI software for measuring Cobb angles, Chen et al. [12] reported an MAE of 2.47°, close to our overall MAE of 2.6°, while Zerouali et al. [13] reported a higher MAE of 2.9° on their predominantly pediatric dataset, compared to our MAE of 1.9° for a similar population. A strength of our DL-based tool is that it is capable of automatically measuring Cobb’s angles with consistently high accuracy and reliability even when there are multiple curvatures and variable severity of scoliosis.

Our study used the whole spine frontal radiographs to measure Cobb angles. While many studies used similar full spine radiographs [1–6], others used frontal chest radiographs to measure thoracic scoliotic curvatures only [7, 14]. While whole spine radiographs are needed for comprehensive scoliosis evaluation, a DL algorithm may be a useful screening tool for incidental scoliosis detection in chest radiographs obtained for other purposes, potentially triggering a need for full spine scoliosis series radiographs.

In comparing the dataset inclusion criteria of our study with other similar studies evaluating Cobb angle measurements, we noted several differences in population demographics, sample size, and data sources. With regard to sample size, our study included a substantial volume of 345 patients, whereas Chen et al. [12] included 108 images and Zerouali et al. [13] included 100 images in their investigations. Furthermore, in their series, Chen et al. [12] studied 108 images from 101 patients, leading to multiple images from the same patient, which could introduce uneven individual data collection and measurement bias. With regard to demographics, our study covered a broader age range of patients, from 4 to 85 years old, while Chen et al. excluded young children and elderly patients (age range of 11 to 64 years). On the other hand, Zerouali et al. included patients from 3 to 64 years but had only nine adults over 18. In terms of data collection, Chen et al. utilized images from two sites and three radiography systems, while Zerouali et al. collected data from four sites and two radiography systems, with 91% of the images sourced from a single site. In comparison, our study utilized data from 5 sites and five different radiography systems, enhancing the representativeness of the data and the generalizability of the results. Although our study had a skewed sex distribution (64% female), this imbalance was more pronounced in Chen et al. (75% female) and similar to Zerouali et al. (65% female). Additionally, with regard to scoliosis severity, Zerouali et al. included only seven patients with severe scoliosis, whereas our study included 24, providing a more statistically robust sample for subgroup analysis. However, it is worth noting that Zerouali et al. included both coronal and sagittal views, which are important for a comprehensive assessment of spinal parameters. The ground truth definition also varied between the studies. In our study, we included two expert musculoskeletal radiologists and one orthopedic spine surgeon, who are the primary professionals involved in Cobb angle measurements and the intended users of the BoneMetrics software. In contrast, Chen et al. and Zerouali et al. relied solely on radiologists for their ground truth definitions. While Chen et al. included more annotators to further mitigate intrareader variability, they lacked a consensus process, potentially excluding the most challenging and complex cases. In Zerouali et al., the ground truth was initially defined by a junior radiologist’s measurements, which were then reviewed and corrected by a senior radiologist.

During manual Cobb angle measurements by human expert readers and subsequent consensus reading, there were some observations about the limitations of our study. One reader consistently underestimated the visual extent of scoliosis and annotated a fewer number of curvatures than the other two readers. In the presence of multiple curvatures in a single patient, it was sometimes difficult for the three readers to fully agree on the locations of the apices of those curvatures. There were a few instances where all three readers disagreed with one another (Fig. 3). When all readers could not agree on one annotation, a majority vote (two-reader agreement) was used as the reference standard. However, one may argue this was arbitrary and subjective. Moreover, there was an asymmetric sex distribution of the subjects (64% women versus 36% men). These demographic characteristics of the included subjects were a source of potential bias in sex- and age-specific measurements of Cobb’s angles. Additional validation using a larger number of older patients can be performed for scoliosis measurements in adult patients. Future work may also include automated measurements of the sagittal balance and lumbopelvic alignments on lateral radiographs and pelvic tilt on frontal radiographs.

Regardless, our findings suggest that a DL-based automated Cobb’s angle measurement tool can perform with high accuracy and reliability compared to human expert readers. This, if it is incorporated into clinical PACS, can offer prospective support to physicians in busy clinical settings. Future studies should investigate the impact on BoneMetrics operational efficiency and reporting times to evaluate its practical benefits in the clinical workflow.

## Data Availability

Not applicable.
